# Intervening in global markets to improve access to HIV/AIDS treatment: an analysis of international policies and the dynamics of global antiretroviral medicines markets

**DOI:** 10.1186/1744-8603-6-9

**Published:** 2010-05-25

**Authors:** Brenda Waning, Margaret Kyle, Ellen Diedrichsen, Lyne Soucy, Jenny Hochstadt, Till Bärnighausen, Suerie Moon

**Affiliations:** 1 Department of Family Medicine, Boston University School of Medicine, One Boston Medical Center Place, Dowling 5 South, Boston, MA 02118, USA; 2 Utrecht University, Utrecht, Netherlands; 3 Toulouse School of Economics, Toulouse, France; 4 National Bureau of Economics, Cambridge, MA, USA; 5 Centre for Economic Policy Research, London, UK; 6 Partners In Health, Boston, MA USA; 7 Boston University School of Public Health, Data Coordinating Center, Boston, MA, USA; 8 Harvard School of Public Health, Department of Global Health and Population, Boston, MA 02115, USA; 9 University of KwaZulu-Natal, Africa Centre for Health and Population Studies, Mtubatuba 3935, South Africa; 10 Sustainability Science Program, Center for International Development, Harvard Kennedy School of Government, Cambridge, MA 02138, USA

## Abstract

**Background:**

Universal access to antiretroviral therapy (ART) in low- and middle-income countries faces numerous challenges: increasing numbers of people needing ART, new guidelines recommending more expensive antiretroviral (ARV) medicines, limited financing, and few fixed-dose combination (FDC) products. Global initiatives aim to promote efficient global ARV markets, yet little is known about market dynamics and the impact of global policy interventions.

**Methods:**

We utilize several data sources, including 12,958 donor-funded, adult first-line ARV purchase transactions, to describe the market from 2002-2008. We examine relationships between market trends and: World Health Organization (WHO) HIV/AIDS treatment guidelines; WHO Prequalification Programme (WHO Prequal) and United States (US) Food and Drug Administration (FDA) approvals; and procurement policies of the Global Fund to Fight AIDS, Tuberculosis, and Malaria (GFATM), US President's Emergency Plan for AIDS Relief (PEPFAR) and UNITAID.

**Results:**

WHO recommended 7, 4, 24, and 6 first-line regimens in 2002, 2003, 2006 and 2009 guidelines, respectively. 2009 guidelines replaced a stavudine-based regimen ($88/person/year) with more expensive zidovudine- ($154-260/person/year) or tenofovir-based ($244-465/person/year) regimens. Purchase volumes for ARVs newly-recommended in 2006 (emtricitabine, tenofovir) increased >15-fold from 2006 to 2008. Twenty-four generic FDCs were quality-approved for older regimens but only four for newer regimens. Generic FDCs were available to GFATM recipients in 2004 but to PEPFAR recipients only after FDA approval in 2006. Price trends for single-component generic medicines mirrored generic FDC prices. Two large-scale purchasers, PEPFAR and UNITAID, together accounted for 53%, 84%, and 77% of market volume for abacavir, emtricitabine, and tenofovir, respectively, in 2008. PEPFAR and UNITAID purchases were often split across two manufacturers.

**Conclusions:**

Global initiatives facilitated the creation of fairly efficient markets for older ARVs, but markets for newer ARVs are less competitive and slower to evolve. WHO guidelines shape demand, and their complexity may help or hinder achievement of economies of scale in pharmaceutical manufacturing. Certification programs assure ARV quality but can delay uptake of new formulations. Large-scale procurement policies may decrease the numbers of buyers and sellers, rendering the market less competitive in the longer-term. Global policies must be developed with consideration for their short- and long-term impact on market dynamics.

## Background

Although much progress has been achieved in scaling-up access to HIV/AIDS treatment in low and middle-income countries, the 4 million people who had received antiretroviral therapy (ART) by the end of 2008 still represent only a small fraction of the 22 million estimated to need treatment by 2015 [1]. Donors provided $10 billion in 2007, but an estimated $50 billion will be required to cover all HIV/AIDS program costs in 2015 [1]. At the same time, new World Health Organization (WHO) guidelines recommend not only using better, more expensive medicine, but also starting ART earlier, implying immediate increases in the numbers of people eligible for treatment [2]. As costs and needs escalate, however, international organizations are facing serious financing shortfalls. For example, in late 2008 the Global Fund to Fight AIDS, Tuberculosis, and Malaria (GFATM) asked principal recipients to decrease eighth-round budgets by 10% [3]. The fallout from the current world economic crisis, meanwhile, is still uncertain. With this "perfect storm" of converging dynamics, policy makers urgently need to understand all factors affecting our ability to meet universal access goals. Market factors, in particular, add even more complexities to the situation.

By intervening in global antiretroviral (ARV) markets serving low- and middle-income countries, the GFATM [4], the Clinton Health Access Initiative (CHAI) [5], the US President's Emergency Plan for AIDS Relief (PEPFAR) [6] and UNITAID [7], among other international organizations, are working to narrow the gap between the funding available and the amounts necessary to achieve universal access. Their interventions aim to provide safe, acceptable and good quality diagnostics and medicines for HIV/AIDS treatment and care, and to promote competition among suppliers. The organizations, however, currently confront daunting challenges and a very different marketplace compared to ART scale-up conditions of the past. Recently available data enable us to describe and assess these changing conditions.

Of pressing concern is the shifting demand for antiretrovirals as countries adopt the newer, more expensive first-line regimens recommended by WHO [2,8]. Some key ARVs in newer regimens are widely patented, while patents for older ARVs were largely absent in the countries that produced and exported them, namely India, Brazil, and Thailand [9]. These and other developing countries now must provide patent protection for more recently-developed medicines as they implement the World Trade Organization (WTO) Agreement on Trade Related Aspects of Intellectual Property Rights [10]. Patent-related barriers for newer regimens result in a less competitive and more fragmented generic market; they also hamper development of improved formulations such as fixed-dose combination (FDC) products, in which two or more medicines are combined into a single tablet. WHO strongly recommends the use of FDCs [8] because of their numerous advantages over single component medicines, most notably simplified prescribing, improved patient adherence, reduced risk of resistance and easier supply chain management [11-15]. Yet far fewer FDCs are available for newer than for older first-line regimens.

Quality assurance and procurement issues also factor into the complex market equation. Initiatives such as the WHO Prequalification Programme (WHO Prequal) [16] and the tentative approval system of the United States (US) Food and Drug Administration (FDA) [17,18] not only ensure that ARVs procured with donor funds meet international quality standards, but also influence the rate and extent of ARV dispersion across low- and middle-income countries. The establishment of large-scale purchasers such as PEPFAR, UNITAID, and the Voluntary Pooled Procurement program of the GFATM, which relieves individual countries of their procurement responsibilities, is rapidly consolidating the number of buyers in the market.

Research to date on ARV markets has focused largely on the evolution of ARV prices [19-23]. Other elements of the "perfect storm" -- in particular the interconnectedness of decisions made by international organizations and their relationships to ARV market dynamics -- have not been well described. Yet understanding these relationships is critical to support future policy making.

To further such understanding, this paper describes the most salient supply- and demand-side characteristics of the market for first-line, adult ARVs in low- and middle-income countries and illustrates relationships between market evolution and the policies of international organizations. We examine ARV market trends in relation to three areas of intervention: WHO HIV/AIDS treatment guidelines; certification decisions of WHO Prequal and FDA; and pooled procurement policies of GFATM, PEPFAR and UNITAID. Since these three factors play out in markets simultaneously, we believe that examining them in relation to one another will provide policy makers and academicians with a more useful analysis than focusing on any one of them in isolation.

## Methods

Using several data sources, we created a dataset of market intelligence information for ARVs that includes purchases made with donor funds in low- and middle-income countries. Information on approvals of quality-assured FDC ARVs was obtained from WHO Prequal [16] and the US FDA [17,18] and added to an analytic dataset that contains ARV product information (manufacturer, strength, dosage form, and price when available) obtained from MSF *Untangling the Web of Price Reductions *[24], CHAI consortium ARV price lists [25], and various manufacturer and national drug regulatory authority websites.

All of this information was used to systematically validate ARV products and prices for ARV purchase transactions obtained from the WHO *Global Price Reporting Mechanism *[26] and the GFATM *Price Quality Report *[27] from 2002-2008, after merging and removal of duplicates.

**Figure 1:**
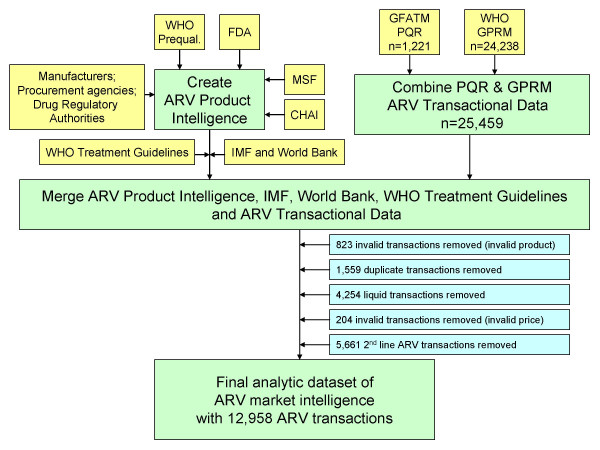
**Description of analytic data set**.

In addition, we included information from the World Bank on country income classifications [28], the International Monetary Fund on annual inflation [29], and WHO on recommended first-line regimens in all editions of WHO adult treatment guidelines for HIV/AIDS [2,8,30,31]. We restricted our analytic dataset to solid dosage forms (tablets, capsules) of adult ARVs used for first-line treatment of HIV/AIDS, namely abacavir (ABC), efavirenz (EFV), emtricitabine (FTC), lamivudine (3TC), nevirapine (NVP), stavudine (d4T), tenofovir (TDF), and zidovudine (ZDV). A detailed process of the creation of the analytic data set is provided in Figure 1.

We adjusted all prices, provided by GFATM and WHO in US Dollars, to the January-December 2008 time period using the annual US Consumer Price Index [29]. We then conducted a descriptive and comprehensive case study on the global market for adult first-line ARVs in low- and middle-income countries.

We present trends from 2002-2009 in the number of first-line regimens recommended by WHO by showing the main regimens that appear in key tables and figures of WHO HIV/AIDS treatment guidelines [2,8,30-32]. We do not include regimens recommended in specific situations as noted throughout the text and footnotes of guidelines. For the purpose of this paper, "older" regimens are defined as those recommended in 2003 WHO Guidelines and "newer" regimens are those in 2006 WHO Guidelines.

Antiretroviral demand is estimated by volumes purchased and presented in person-years whereby:

When estimating volume of ARVs purchased, we include all products (FDCs, co-packaged products, and individual medicines) that contain the ARV of interest in calculating volumes purchased. For example, the total volume purchased for tenofovir would include TDF, 3TC/TDF, FTC/TDF, and EFV/FTC/TDF.

Antiretroviral prices are calculated using adult dosages for persons weighing greater than sixty kilograms [8], whereby:

Median prices plus 25^th ^and 75^th ^percentile prices are provided for the most commonly used first-line ARV regimens [33] and calculated using the least expensive ARVs to create each regimen. For example, the stavudine (d4T) 30, lamivudine (3TC) 150, nevirapine (NVP) 200 regimen price is based upon the price of the generic fixed-dose combination product, whereas the tenofovir (TDF) 300, emtricitabine (FTC) 200, NVP200 regimen is based upon generic prices of TDF300/FTC200 fixed-dose product and NVP200 tablet.

For three-in-one FDCs, we plot timelines of products and manufacturers approved by the FDA approval, FDA tentative approval, and WHO Prequalification systems from 2000-2009 [16-18].

In depicting FDC market dynamics, for each year we present the number of manufacturers reported in transactional purchase data, the total number of manufacturers who have been approved by either WHO Prequal or US FDA to date, and the number of countries who purchased the FDC.

We describe FDC products using a "/" between ARVs included in a given FDC. We use a "+" to depict regimens comprised of two or three distinct tablets. For example, for the regimen of 3TC150, NVP200, and ZDV300, the format 3TC150/NVP200/ZDV300 reflects the FDC version, whereas 3TC150+NVP200+ZDV300 reflects three individual tablets, and 3TC150/ZDV300 + NVP200 reflects a FDC plus an individual NVP200 tablet.

We present trends in market share by volume for the most commonly used three-in-one FDCs by plotting the annual volume (in person-years) bought by each purchaser. The purchaser is defined as the organization providing funds to buy ARVs and includes four categories: GFATM, PEPFAR, UNITAID and miscellaneous. The PEPFAR purchases are actually purchases made by the Supply Chain Management System (SCMS), a consortium organization that purchases ARVs on behalf of PEPFAR. In our data sources, no PEPFAR purchases were recorded outside of SCMS. The manufacturer split across each purchaser is also depicted.

2008 market share is calculated across purchasers according to both the value (in US Dollars) and the volume (in person-years) of ARVs purchased. Analyses of 2008 market share include all products (FDCs, co-packaged medicines, and individual medicines) that contain the ARV of interest.

## Results

### Relationships between WHO treatment guidelines and demand

**Figure 2:**
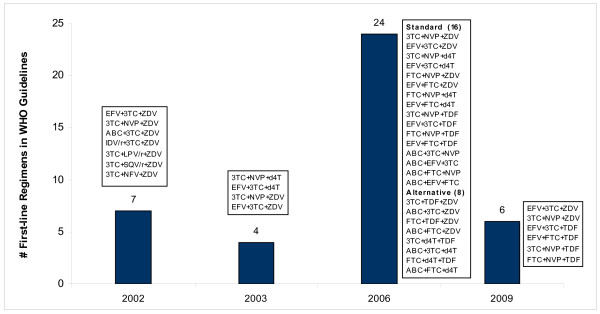
**Trends in numbers of 1^st ^line ARV regimens in WHO treatment guidelines**.

Figure 2 shows the composition of WHO treatment guidelines from 2002-2009. The number of first-line regimens and their components varied significantly, with corresponding swings in purchase volumes, as described below in more detail.

The first WHO HIV/AIDS treatment guidelines for adults and adolescents were released in 2002. They recommended seven regimens comprised of ten ARVs, including the relatively costly protease inhibitors (Figure 2) [30]. One year later, WHO issued revised guidelines that included only four key first-line regimens [31] comprised of five different ARVs, namely EFV, 3TC, NVP, d4T and ZDV; these guidelines excluded protease inhibitors altogether [31].

In 2006, WHO released a second revision of HIV/AIDS treatment guidelines [8] with an increase to 24 recommended first-line regimens (16 regimens characterized as "standard" and eight characterized as "alternative") [8]. The revision offered much more flexibility in terms of clinical options for prescribers. To the five ARVs in the 2003 guidelines, the 2006 revision added three more, namely ABC, FTC, and TDF. The 2006 guidelines also suggested that practitioners start planning to move away from d4T-based regimens due to related toxicities [8]. In May 2007, WHO issued an addendum recommendation to dose d4T at 30 mg twice daily for all adults regardless of weight, replacing the previous dosing of 40 mg twice daily for patients weighing more than 60 kilograms [32].

The latest WHO revisions, announced in November 2009 and to be officially released in 2010 [2], recommend only six key first-line regimens comprised of six ARVs for treatment-naïve individuals [2]. Each of these regimens contains ZDV or TDF plus 3TC or FTC plus EFV or NVP [2]. The 2009 regimens do not introduce new ARVs or regimens, but prioritize regimens listed in the 2006 guidelines. The newest guidelines no longer recommend the use of d4T because of its side effects and toxicities.

**Figure 3:**
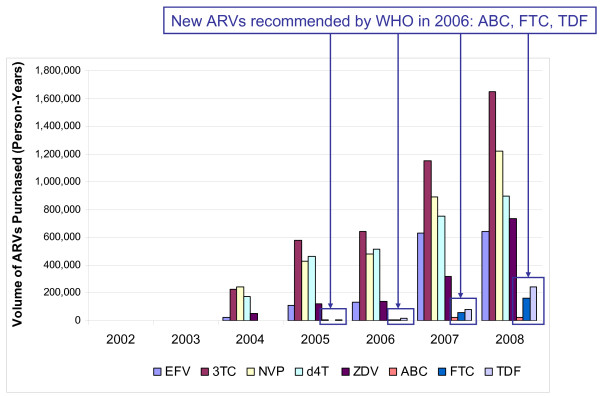
**Consumption trends of WHO-recommended first-line ARVs (2002-2008)**.

Examination of purchase trends for first-line ARVs strongly suggests that the WHO guideline recommendations play an important role in driving ARV demand. The five ARVs listed in the 2003 WHO treatment guidelines accounted for more than 98% of ARVs purchased in 2004-2006 (Figure 3). Shortly after the addition of TDF and FTC to WHO first-line treatment guidelines in 2006, TDF purchase volumes increased more than 15-fold, from 16,000 person-years in 2006 to 240,000 person-years in 2008, while FTC purchase volumes increased more than 20-fold over the same period, with 162,000 person-years of purchase volume noted in 2008.

Similarly, purchase patterns appear to reflect 2006 WHO guidance away from d4T-containing regimens [8]. From 2006 to 2008, demand for d4T increased less than two-fold from 515,000 person-years to 895,000 person-years, while demand for ZDV (the lowest-cost substitute for d4T) grew more than five-fold, from 139,000 person-years to more 733,000 person-years over the same time period.

### Price implications of new WHO Guidelines

Prices for newer first-line regimens (those more recently recommended by WHO) are considerably higher than prices for older regimens. In 2008, the most commonly used older regimen (3TC+NVP+d4T) was $88/person/year in low-income countries. As countries adopt new 2009 WHO recommendations to phase out d4T use, they are likely to instead use ZDV-based regimens priced 1.8-3 times higher at $154 (3TC/NVP/ZDV) and $260 (EFV+3TC/ZDV) or a TDF-based regimen (TDF+3TC+NVP), priced 2.8 times higher at $244/person/year in low income countries (Table 1).

**Table 1:** 2008 Prices for most-commonly used first-line ARV regimens

	Median (25^th^, 75^th ^percentile) Regimen Prices* in USD		
	**Low** **Income**	**Lower-Middle** **Income**	**Upper-Middle** **Income**

**Old First-Line Regimens from 2003 WHO Guidelines:**			
3TC/NVP/d4T30	88 (83, 90)	87 (80, 151)	110 (84, 222)
EFV+3TC/d4T30	198 (183, 223)	147 (52, 253)	211 (172, 235)
3TC/NVP/ZDV**	154 (144, 162)	172 (154, 259)	161 (161, 189)
EFV+3TC/ZDV**	260 (246, 286)	216 (118, 298)	326 (260, 370)
			

**New First-Line Regimens from 2006, 2009 WHO Guidelines:**			
3TC+NVP+TDF**	244 (226, 278)	256 (244, 288)	387 (311, 591)
EFV+3TC+TDF**	349 (321, 399)	301 (207, 392)	477 (404, 527)
FTC/TDF+NVP**	361 (325, 366)	399 (292, 427)	525 (368, 726)
EFV+FTC/TDF**	465 (419, 487)	443 (256, 531)	616 (461, 663)
ABC+3TC+NVP	398 (361, 450)	418 (392, 457)	491 (443, 705)
ABC+EFV+3TC	503 (455, 571)	463 (355, 561)	581 (536, 641)
ABC+FTC+NVP	n/a^§^	n/a^§^	n/a^§^
ABC+EFV+FTC	n/a^§^	n/a^§^	n/a^§^

*price/person/year calculated using the least expensive ARVs to create each regimen (see methods section)

**first-line regimens recommended in 2009 WHO guidelines

^§^price data unavailable; less than 5 purchases for at least one ARV in regimen

### Relationships between regulatory bodies and availability of ARV FDCs across donor programs

WHO established WHO Prequal in 2001 to ensure that medicines purchased with funds from United Nations organizations met international quality standards [16]. In most cases, principal recipients of GFATM funds are required to purchase medicines pre-qualified by WHO Prequal or strict regulatory authorities such as the US FDA, the European Medicines Agency, or Health Canada.

The US FDA established the tentative approval system in May 2004 to enable PEPFAR recipients to access generic versions of products still under patent protection or other forms of market exclusivity in the US and to expedite approval of ARVs [17]. Antiretroviral medicines purchased with PEPFAR funds must be approved by either the standard or the tentative FDA approval process [17].

**Figure 4:**
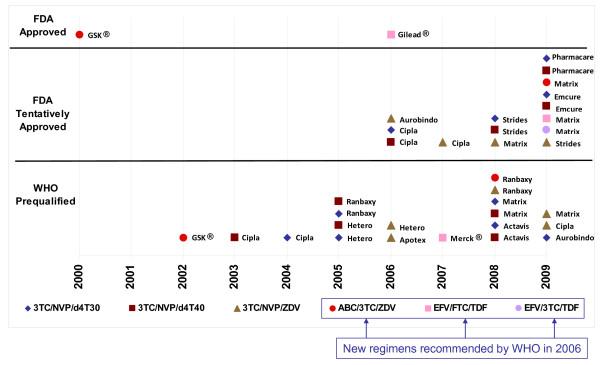
**Timeline of WHO Prequalification Programme and US FDA approvals of first-line fixed-dose combination ARVs**.

Figure 4 illustrates the timing of regulatory approval for different WHO-recommended FDCs. By the end of 2009, 19 three-in-one FDCs had been approved through WHO Prequal and 15 FDCs through the FDA tentative process. The first generic FDC (3TC/NVP/d4t40) was prequalified by WHO in 2003 (Figure 4), the same year WHO released guidelines recommending use of the FDC as one of four regimens. By 2006, six d4T-based FDCs and two ZDV-based FDCs were WHO-prequalified. In contrast, the FDA first approved a generic FDC (3TC/NVP/ZDV) in mid-2006 (thereby allowing PEPFAR programs to purchase them), approximately three years after the release of 2003 WHO Guidelines. The FDA first approved d4T-based FDCs in November 2006, approximately three years after the first approval by WHO (Figure 4). In short, the FDA approved FDCs for older regimens several years after WHO, which was reflected in delayed market demand from PEPFAR recipients for these products.

Quality-assured generic FDC ARVs used in newer regimens are appearing at a much slower rate than that observed with older regimens. While 24 generic FDCs have been approved by either FDA or WHO to support older regimens recommended in 2003, only four generic FDCs have been approved to support new regimens recommended by WHO in 2006: two ABC-based FDCs no longer prioritized on 2009 WHO guidelines, and two TDF-based FDCs. Three of these were approved through the tentative FDA process and only one through WHO Prequal.

### Relationships between prices of three-in-one FDC ARVs and their component medicines

**Figure 5:**
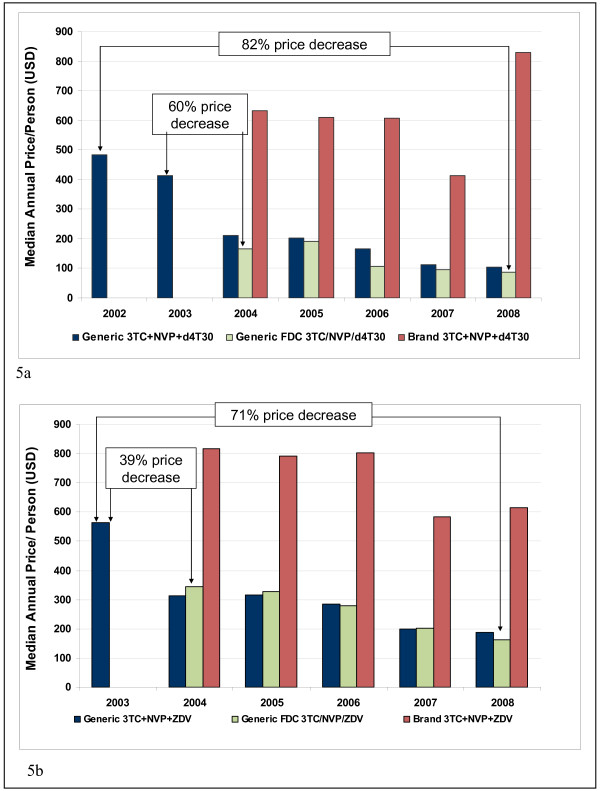
**Price trends for three-in-one FDCs and their component medicines**. 5a. Price trends for 3TC, NVP, and d4T30. 5b. Price trends for 3TC, NVP, and ZDV.

Prices for older ARV regimens have decreased dramatically over the past seven years. For the 3TC, NVP, and d4T30 regimen, the median price when purchasing three generic, single-ingredient ARVs was $484/person/year in 2002 and decreased 82% by 2008 to $88/person/year when purchasing the generic FDC (Figure 5a). The ZDV-based regimen of 3TC, NVP, and ZDV exhibited the same trends with the median price for three generic, single-ingredient ARVs decreasing 71% from $564/person/year for the three generic, single-ingredient ARVs in 2003 to $161/person/year in 2008 for the generic FDC (Figure 5b).

All regimens, including those provided through single ingredient medicines, copackaged medicines, and FDCs, exhibit steep price reductions upon market entry of the generic FDC. Price reductions of 60%, 66% and, 39% are noted when the FDC version first appear compared to prices for three single-ingredient ARVs in the previous year for d4T-30, d4T-40, and ZDV-based regimens, respectively (Figure 5a and Table 2).

**Table 2:** Price trends for first-line, three-in-one FDCs and their component ARVs

	Median (25^th^, 75^th ^percentile) Regimen Prices* in USD						
	**2002**	**2003**	**2004**	**2005**	**2006**	**2007**	**2008**

**3TC, NVP, d4T40**							

Generic NVP+3TC+d4T40	490 (486, 496)	418 (245, 489)	212 (184, 249)	209 (183, 255)	169 (150, 172)	114 (108, 130)	107 (97, 149)

Brand NVP+3TC+d4T40			640 (640, 648)	619 (619, 707)	618 (597, 746)	637 (370, 954)	897 (601, 1,219)

Generic FDC		165*	180 (143, 193)	180 (163, 214)	112 (112, 129)	83 (83, 102)	104 (80, 151)

							

**ABC, 3TC, ZDV**							

Generic ABC+3TC+ZDV			1,083 (510, 1591)	1,101 (1,039, 1,212)	794 (744, 813)	568 (525, 626)	475 (436, 587)

Brand ABC+3TC+ZDV		1,669*	1,329 (1,329, 1,363)	1,286 (1,285, 1,387)	1,282 (978, 1,354)	984 (938, 991)	702 (681, 1,064)

Brand FDC		1,652*	1,483*	1,366 (1,366, 1,489)	1,363*	883 (883, 989)	
							

**EFV, FTC, TDF**							

Generic EFV + FTC/TDF						516 (417, 536)	464 (441, 487)

Brand EFV + FTC/TDF				781*	678 (636, 769)	593 (579, 624)	619 (573, 834)

Generic FDC							485*

Brand FDC						712*	613*

*25^th ^and 75^th ^percentiles not calculated because n < 5 purchases

Generic prices for the three single ingredients mirror prices of FDCs after their launch. Whereas d4T-based FDCs offer consistent price discounts compared to their components, the ZDV-based FDC entered at a slightly higher price than its components but by 2008 offered savings. Prices for single-ingredient, branded ARVs consistently ranged from 2.4-9.5 times higher than prices for generic FDCs for both d4T- and ZDV-based regimens.

For newer regimens recommended by WHO in 2006, only two FDCs were purchased: ABC/3TC/ZDV and EFV/FTC/TDF. No generic version of the ABC-based FDC was purchased and prices for the branded FDC were consistently higher compared to prices for the three generic ARVs (Table 2). Similarly, the branded TDF-based FDC with EFV offers no price savings over purchasing three generic ARVs (Table 2). A generic EFV-based FDC was first reported in 2008 and its price is similar to the price of three generic ingredients.

### Market dynamics for three-in-one FDC ARVs

The market dynamics of FDC versions of ARVs are indicative of market efficiency over the past several years, at least using typical measures of competition. First, there has been a large increase in the number of manufacturers. In addition, the number of purchasers and total volume purchased increased. A reduction in the market power of suppliers has likely contributed to the reduction in price, while at the same time the increases in demand have attracted new entry by generics producers.

**Figure 6:**
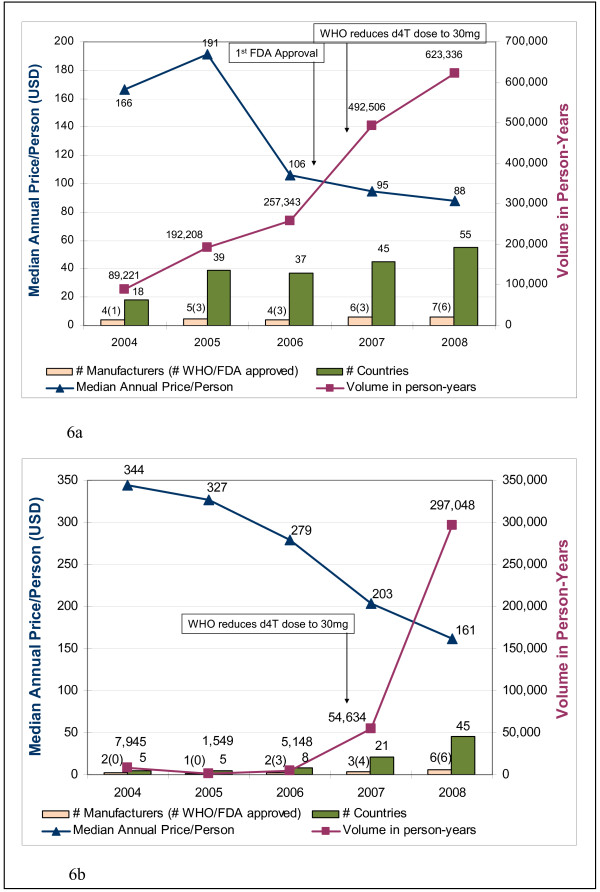
Market dynamics for three-in-one FDCs*. * # of manufacturers refers to the number of manufacturers who sold ARVs to donor recipients in a given year, as reported to either GFATM or WHO; this is NOT the total # of manufacturers in the market. 6a. Market dynamics for lamivudine/nevirapine/stavudine 30 FDC. 6b. Market dynamics for lamivudine/nevirapine/zidovudine 30 FDC.

For the 3TC/NVP/d4T30 FDC, the number of manufacturers approved by WHO or FDA increased from one to six from 2004 to 2008, while the number of manufacturers who sold this FDC to recipient countries increased from four to seven over the same time period (Figure 6a). By 2008, 55 countries were purchasing this FDC. An increase in purchase volume makes entry more attractive to new suppliers and may also facilitate economies of scale in production. Purchase volume rose dramatically from 2004 to 2008, from 89,221 to 623,336 person-years. Notable increases in purchase volume occurred for this FDC following the first FDA approval in December 2006. More striking, though, is the immediate reaction to the WHO recommendation to reduce d4T dosing from 40 mg to 30 mg in May 2007. Purchase volumes for the 40 mg d4T-based FDC immediately dropped off (Table 3), while purchase volumes for the 30 mg d4T-based FDC sharply increased (Figure 6a). As purchase volumes increased for 3TC/NVP/d4T30 FDC, the global median price decreased from $166/person/year in 2004 to $88/person/year in 2008.

**Table 3:** Market dynamics for FDC versions of 3TC/NVP/d4T40, ABC/3TC/ZDV and EFV/FTC/TDF

	2004	2005	2006	2007	2008
**3TC/NVP/d4T40:**					

# Manufacturers *	2	5	3	4	3

# Approved Manufacturers (WHO & FDA)	1	3	3	3	6

# Countries	21	38	32	21	7

Volume in person-years	55,758	126,005	114,178	103,690	14,810

Median annual price/person, USD (generic)	180	180	112	83	104
					

**ABC/3TC/ZDV:**					

# Manufacturers*	3	2	1	1	3

# Approved Manufacturers (WHO & FDA)	1	1	1	1	2

# Countries	3	6	2	3	2

Volume in person-years	61	250	106	479	129

Median annual price/person, USD (brand)	1,483	1,366	1,363	883	3,257
					

**EFV/FTC/TDF:**					

# Manufacturers*				1	3

# Approved Manufacturers (WHO & FDA)			1	2	2

# Countries				1	7

Volume in person-years				335	3,720

Median annual price/person, USD (brand)				712	613

* # of manufacturers refers to the number of manufacturers who sold ARVs to donor recipients in a given year, as reported to either GFATM or WHO; this is NOT the total # of manufacturers in the market

Market dynamics around the 3TC/NVP/ZDV FDC are similar. From 2004 to 2008, the number of manufacturers approved by WHO or FDA increased from zero to six, while the number of manufacturers who sold the medicine to recipient countries increased from two to six (Figure 6b). Similar purchase volume increases were noted for the ZDV-based FDC which is often used in place of d4T (Figure 6b) immediately after the 2007 WHO guidance to reduce d4T dosing.

Market dynamics for 3TC/NVP/d4T40 were similar to those already described except for dramatic decreases in purchase volume noted after WHO issued guidance recommending lower doses of d4t. While purchase volumes had grown to more than 100,000 person-years in 2007, they decreased to fewer than 15,000 person-years in 2008 (Table 3).

Analysis of FDC market dynamics for newer regimens reveals relatively low purchase volumes and higher prices as compared to FDCs used in older regimens. While the branded ABC/3TC/ZDV FDC was FDA-approved in 2000 (Figure 4), demand for this product has been low, peaking at fewer than 500 person-years of volume in 2007 but dropping dramatically thereafter (Table 3). The branded EFV/FTC/TDF was FDA-approved in 2006 (Figure 4), but demand for the FDC has only just started to grow, reaching 3,720 person-years of volume in 2008.

### Trends in FDC market share across purchasers and manufacturers

**Figure 7:**
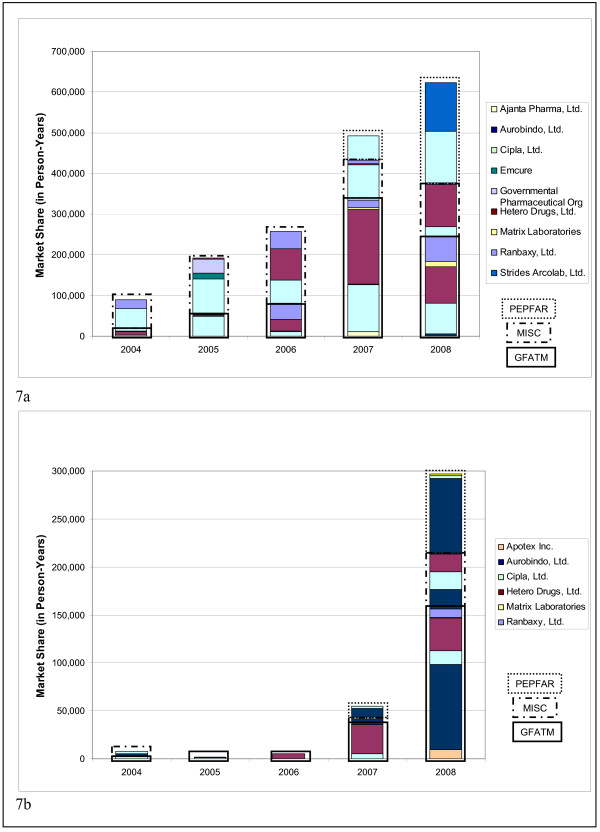
**FDC Annal market trends by purchaser and manufacturer**. 7a. Market trends for 3TC/NVP/d4T30 by purchaser and manufacturer. 7b. Market trends for 3TC/NVP/ZDV by purchaser and manufacturer.

Analysis of market share by both purchasers and the manufacturers that supply them reflects the dominant role large-scale buyers are beginning to play in the global market. PEPFAR was the first large-scale purchaser and it changed the market structure for first-line FDCs. The first FDC version of 3TC/NVP/d4T30 was only approved by the FDA tentative approval system in November 2006 (Figure 4), allowing PEPFAR to begin purchasing in 2007. For 2004-2006, therefore, GFATM was the major purchaser and the market was split across the various manufacturers chosen by the principal recipients of GFATM funds. By 2008, however, PEPFAR, represented 40% of the total market for this FDC, with purchases split across only two manufacturers (Figure 7a).

The same general trends are observed with the FDC version of 3TC/NVP/ZDV. By 2008, PEPFAR accounted for 28% of market volume for this product, with purchases split across three manufacturers, one of which accounted for 94% of PEPFAR purchases (Figure 7b). In contrast, the GFATM's disaggregated purchases for both these FDCs are split across 4-5 different manufacturers.

### Cross-section of 2008 market share by purchaser for all ARVs containing first-line medicines

The impact of large-scale purchasing organizations on market dynamics - both market value and market volume -- is even more pronounced in analyses on all ARVs (single-ingredient, co-packaged medicines, and FDCs) containing first-line medicines.

**Figure 8:**
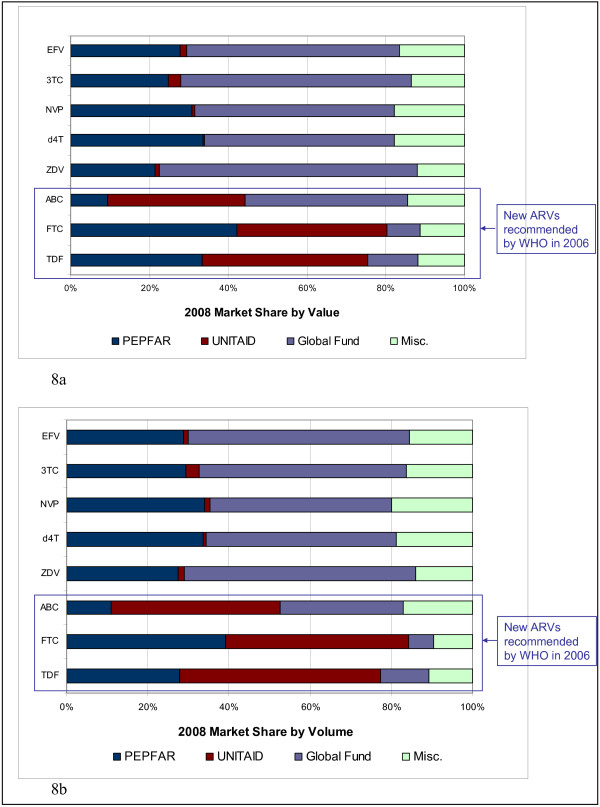
**2008 Market share across purchasers**. 8a. 2008 Market share by value across purchasers. 8b. 2008 Market share by volume across purchasers.

For newer first line ARVs recommended by WHO (ABC, FTC and TDF), PEPFAR accounts for 9%, 42%, and 33% of market value, respectively, while UNITAID accounts for 35%, 38%, and 42%, respectively (Figure 8a). Indeed, PEPFAR and UNITAID together account for 44%, 80% and 75% of the global market for ABC, FTC and TDF, respectively, while the GFATM accounts for 41%, 8%, and 13% (Figure 8a).

Examination of purchaser market share by volume reveals similar results. For older first- line ARVs (EFV, 3TC, NVP, d4T, and ZDV), PEPFAR accounts for 27-34% of market by volume, while the GFATM accounts for 47-57% (Figure 8b).

For the newer first line ARVs (ABC, FTC, and TDF), PEPFAR accounts for 11%, 39%, and 28% of market volume, respectively, while UNITAID accounts for 42%, 45%, and 49%, respectively (Figure 8b). Again, PEPFAR and UNITAID together account for 53%, 84% and 77% of the global market for ABC, FTC, and TDF, respectively, while the GFATM accounts for only 30%, 6%, and 12%, respectively (Figure 8b). It is worth noting that many of these ARVs can be used in both first- and second line regimens and that the majority of UNITAID purchases are likely used in second line treatment. Regardless of whether these ARVs are used for first- or second line treatment, PEPFAR and UNITAID clearly dominate the market for these products.

## Discussion

### Relationships between interventions and markets

The data presented here strongly suggest that the policies of donors and international organizations bear directly on the evolution of antiretroviral medicines markets in low- and middle-income countries. A number of highlights emerge in our analyses.

#### 1. Remarkably efficient ARV markets evolved shortly after the 2002 establishment of the GFATM for the most commonly used first-line ARVs

The entry of many manufacturers producing quality-assured generic ARVs, dramatic price reductions, and the development of innovative FDCs all indicate a largely decentralized and efficient market by conventional measures. Purchase arrangements likely contributed to fierce competition among producers of older ARVs, as GFATM funding was distributed to more than 100 countries that each made independent purchase decisions. Disaggregated purchasing promoted competition for products and geographic market niches among producers. The absence of blocking patents in India, where most of the generic ARVs are produced, and efforts by importing governments to overcome patent barriers also contributed to a competitive, efficient global market for older ARVs.

#### 2. The roles of WHO and the FDA have had mixed effects on development and uptake of new ARV formulations

*Quality Certification*: WHO Prequal and the FDA maintained a level playing field by assuring that producers were competing on ARVs of similar quality, and corrected information asymmetries around quality for purchasers. However, delays in quality certification can also create delays in country uptake of products, as demonstrated by the three-year wait to use PEPFAR funds for the most commonly-used FDC (3TC/NVP/d4T). These programs must perform at optimal efficiency to support the timely maturation of global ARV markets.

*Treatment Guidelines*: WHO also exerted substantial leverage in dictating demand for certain ARVs through its HIV/AIDS treatment guidelines. The 2003 guidelines consolidated demand around four first-line regimens, which covered 94% of people on ART as of 2006; 80% were on regimens available as generic FDCs [33]. Such consolidation of demand created incentives for manufacturers to enter the generic ARV market and develop innovative FDC products to support these regimens. In contrast, WHO's 2006 guidelines listed more than 20 first-line regimens. This increase in treatment options, among other factors, may have created disincentives for manufacturers to develop FDCs of the newer regimens. The 2009 WHO Guidelines have now come full circle, reducing the number of recommended first-line regimens to six, which may again facilitate the consolidation of demand around a few ARVs and encourage manufacturers to produce and develop FDCs of the newly recommended regimens.

#### 3. Newly recommended WHO ARVs are much more expensive due to patent status and/or immature generic markets, creating concern for countries' abilities to adopt new guidelines

Generics markets for newer ARV regimens have not yet matured, as demonstrated by high prices, low demand, small numbers of manufacturers, and only a few three-in-one FDCs. Of particular concern are regimens that include newer ARVs such as tenofovir, which are priced at least 3 times more than older regimens. In the absence of measures to decrease drug prices and/or increase funding, countries may be forced to choose between treating fewer people with newer and "better" regimens or treating more people with older and "less desirable" regimens.

To date, generic competition has been the only proven method to promote sustained and substantial price reduction. However, increasing implementation of the TRIPS Agreement in developing countries means that medicines patents are becoming more widespread and severely restricting or eliminating generic competition for newer ARVs.

Such patents could severely restrict or eliminate generic competition. Least developed countries, however, have a waiver from TRIPS obligations on pharmaceutical patents and data protection until 2016.

Gilead has offered voluntary licenses to multiple firms to produce tenofovir, which has enabled competition in production. Many of these licenses include restrictions - for example, regarding API sources and eligible export markets [34]. It is too soon to evaluate any impacts of theses restrictions.

For those ARVs which are widely patented, additional interventions beyond voluntary licensing will be needed to address intellectual property barriers in both importing and exporting countries. TRIPS flexibilities such as compulsory licensing, non-observation of pharmaceutical patents (allowed for least-developed country WTO members until at least 2016), application of high standards of patentability in national law, and patent pools will be needed to promote market efficiency, reduce prices, and facilitate the use of new FDCs. Such measures have been employed with success in a growing number of countries, but still remain under-utilized relative to need [9,34]. Particularly for essential medicines such as those included in the WHO guidelines, governments, international organizations and other relevant actors should ensure that patent barriers do not stand in the way of widespread, equitable access.

#### 4. Fixed-dose combinations may have been determinants of market prices for their component ARVs

Generic three-in-one FDCs -- strongly recommended by WHO -- were introduced in 2003 at prices much lower than the sum of their generic ARV component prices the previous year. It is possible that manufacturers priced some FDCs aggressively to gain market share and, therefore, created new benchmarks for pricing the component medicines (in addition to other factors such as volume, economies of scale, and robust competition). If so, FDCs may exert a positive influence on ARV markets, above and beyond their public health or logistical advantages.

#### 5. Large-scale purchasing initiatives, including pooled procurement, have transformed some disaggregated markets into consolidated markets comprised of a few key purchasers and the manufacturers they choose to supply their ARVs

PEPFAR and UNITAID have increasingly used pooled procurement, whereby Western third-party organizations purchase medicines on behalf of funding recipients, pooling ARV volumes of several countries into larger, fewer transactions. In 2008, UNITAID and PEPFAR together accounted for 84% of the global market for FTC and 77% for TDF (Figure 7). Meanwhile, both PEPFAR and UNITAID have usually contracted with two or three manufacturers and awarded the majority of their purchases to one or two. The chosen manufacturers then typically dominate the market. These procurement policies may discourage other producers from incurring the costs to develop and produce quality-assured ARVs, thereby decreasing the number of competitors in the market.

The GFATM's Voluntary Pooled Procurement (VPP) program will introduce yet another large-scale purchaser that will further consolidate the number of buyers. Whereas the original design of the GFATM placed medicine procurement in the hands of national principal recipients, VPP will encourage them to pool ARV volumes through third-party procurement. To date, third-party operators involved in VPP include the Supply Chain Management System (SCMS), which conducts pooled procurement for PEPFAR, and CHAI, which handles pooled procurement for UNITAID. If these arrangements persist, SCMS and CHAI will be purchasing on behalf of all the major donors. In this case, the market will no longer be a disaggregated and heterogeneous "open" market of more than one hundred national-level buyers, but instead will be concentrated around a few large-scale purchasers.

Pooled procurement is attractive to donor organizations and governments for a number of reasons. Some organizations may have superior information about supplier costs, the benefits of which (e.g. lower prices), can be shared with others through pooling. Pooled procurement may also reduce overall transaction costs, since fewer transactions occur. If economies of scale are very important at the transaction level, these are more likely to be realized through a few very large transactions rather than many small ones. Pooled procurement might be especially attractive to governments of smaller countries with minimal procurement capacity and limited powers to negotiate with suppliers; it might also be viewed as a solution in countries with documented corruption in procurement.

In practice, however, these benefits may not be realized. Pooled procurement requires the harmonization of registration, intellectual property policies, ARV selection, and demand forecasting across countries and organizations, which can entail substantial coordination costs. Other transaction costs are associated with financial transfers and currency fluctuations. In addition, because pooled procurement is usually handled by staff in developed countries, with higher salaries and overhead, administrative costs may not be lower. Pooled procurement may also lead to dependence by low- and middle-income countries on outside parties, detracting from efforts to strengthen country health systems and build capacity.

The ultimate goal of all these programs is to improve public health. While impossible to determine from this analysis, it is likely that the market approach that best serves public health is a mixture of several different procurement strategies as observed with earlier WHO-recommended 1^st ^line ARVs. In this scenario, the large purchasers such as PEPFAR could drive global prices lower but there was still sufficient purchase power remaining in Global Fund countries to facilitate competition among manufacturers who did not win the larger PEPFAR contracts. A completely disaggregated market may not yield the lowest possible prices while a completely pooled market will likely reduce the number of producers in the long term.

#### 6. For efficient ARV markets, short-term gains must be balanced with long term goals

Global health initiatives are under considerable pressure to document their impact and success. However, for organizations charged with intervening in markets, the indicators for success are not necessarily clear. Examples of short-term goals for market-based initiatives might include ARV price reduction and the development of improved formulations; however, reaching these goals is not necessarily synonymous with building efficient global ARV markets.

In addition, a focus on short-term gains may prove detrimental to market evolution in the long run. The global market is evolving towards greater concentration on the demand side, with the emergence of a few large-scale purchasers, who in turn are encouraging greater concentration on the supply side, by granting tenders to only a few dominant manufacturers. While the immediate effect of lower ARV prices obtained through initiatives such as pooled procurement is attractive, the long-term impact on market efficiency remains a concern. Generally speaking, markets with only a few buyers and suppliers are characterized by both monopoly and monopsony power, and generally function less efficiently. For example, manufacturers may try to offset the discounts they offer large-scale purchasers by increasing prices charged to countries not included in these large-purchase schemes. Demand outside of the large-purchase schemes may be too low to sustain the existing manufacturers and may discourage new ones. Markets dominated by a few manufacturers are more vulnerable to price-fixing and collusion.

#### 7. Conventional market analysis tools may be inadequate for assessing markets and the effects of interventions on ARV markets because these markets are complex and changing rapidly

Systematically identifying market failures and assessing market competition are themselves complex tasks. In theory, perfectly competitive markets exhibit a sufficient number of suppliers and purchasers with perfect information on products; comparable product quality across suppliers; and, freedom from barriers to market entry or exit. In this theoretical scenario, resources are allocated efficiently and competition between suppliers results in lower costs [35]. In practice, however, there is no consensus on definitions or characteristics of a well-functioning market, even by the international community now committed to improving global markets as a means of increasing access to treatment. As noted by the National Academies' Committee on the Economics of Antimalarial Drugs in 2004, "[t]here are no firm rules for judging 'good' prices, or 'healthy' competition." [36].

In addition, standard tools to assess competitive markets are inappropriate in contexts where it is crucial to have dynamic efficiency - i.e., to maintain incentives for continued innovation, quality improvements and development of new treatments. In the short run, perfect competition between suppliers that results in prices close to marginal cost creates static, not dynamic, efficiency; in this case, no supplier expects to profit from additional investment in research and development for new medicines or formulations. Sustainable prices, on the other hand, are those that exceed the marginal cost of production, allowing suppliers to earn a return on research and development investments and creating incentives for additional innovation.

A truly efficient ARV market might, therefore, offer not the lowest prices *per se*, but the lowest prices possible *while at the same time *ensuring continued innovation of quality products in optimum formulations. Price is undeniably an important factor in access, and lower prices enable greater access for the same level of funding. However, a narrow focus on price alone may drive prices to lowest acceptable levels for manufacturers and leave no additional funds to invest in the development of pediatric formulations, FDCs, heat-stable ARVs, and other formulation improvements. Driving prices too low could also create disincentives for manufacturers to enter or remain in the market, especially smaller manufacturers who are unable to shift costs across multiple product lines. Lastly, a focus on price without consideration for supplier performance (e.g., ability to provide the desired amount of medicines in a timely manner, may result in lower prices but increased stock-outs due to sub-par distribution services. Similarly, effective quality assurance systems must be in place in order for ARV markets to deliver the desired health outcomes.

### Limitations and areas for further research

This study provides a comprehensive overview of global policies and ARV market trends suggesting certain causal relationships, but our descriptive methods cannot ascertain causality or pinpoint the impact of a given intervention on the market.

We limit this paper to relationships between a few global initiatives and market trends and do not incorporate the potential market impact of many other key players, including HIV/AIDS activists, civil society organizations, national governments, foundations, and other international organizations. In addition, our data does not capture 100% of the market but rather include only ARV procurements reported to GFATM and WHO, the majority of which are funded by GFATM, PEPFAR, and UNITAID. A few larger, middle-income countries - notably Brazil, South Africa, and Thailand (accounting for 26% of people on ART in the developing world [1]) - purchase large amounts of ARVs with a mix of national and international funds, and do not report their national purchases to the GFATM or WHO. Based on publicly-available information, we estimate that our data capture 27% of purchases from Brazil, South Africa, and Thailand and therefore represent the vast majority of ARV purchases in developing countries. Ideally we would incorporate national ARV purchase data to better understand the important roles these countries play in shaping the global ARV market. For example, some have suggested that Brazil's purchase of active principle ingredients (APIs) and domestic production of ARVs facilitated competition and price reduction for both APIs and ARVs in donor-funded markets [9]. To understand these impacts more clearly, we would need additional purchase data for both APIs and ARVs in these key countries; we encourage national governments to provide their purchase data to the WHO *Global Price Reporting Mechanism *in order to enable improved understanding of and policy interventions in global ARV markets.

We furthermore recognize certain limitations with regards to the quality and reliability of source data. The ARV transactional data, in particular, required substantive cleaning. While we believe we have done due diligence by scrutinizing, systematically cleaning, and validating every transaction, some reporting errors may still exist.

In addition, we note the disappearance of historical transactional data that had previously been posted by WHO and GFATM. For this paper, we used 2002-2008 purchases downloaded from the WHO and GFATM on 1 June, 2009 and 1 September, 2009, respectively; but observed that some historical transactions we were able to download on earlier dates were not present in the downloaded data we used for this paper. Similarly, we noted differences in dates and ARVs listed on various updates of FDA approval and WHO Prequal lists and use information downloaded from these two organizations on 3 January, 2010.

Due to the absence of comprehensive, reliable, publicly-available data on patents and other intellectual property barriers in many low- and middle-income countries, we were unable to include this information in our analyses. We recognize the importance of national policies and registrations in market evolution, but had no access to this information. We lacked access to market intelligence for active principle ingredients, intermediates, and production costs; we also have no information on the use of wholesale procurement agencies. In the discussion section we hypothesize about aggressive pricing and incentives/disincentives for development of FDCs by manufacturers, but we did not conduct interviews with manufacturers to confirm our speculations.

Despite these limitations, our research provides valuable insight for those working to promote market efficiency in order to increase access to ARVs. This paper lays out the first logical steps toward better understanding the many ways that initiatives of international organizations affect ARV markets, and can be used to inform basic monitoring and evaluation systems of those organizations involved with market dynamics. Many organizations now routinely compile market intelligence data, but it needs to be made publicly available in reliable, synchronized and ready-to-use formats to support day-to-day procurement, decision making, and evaluation of interventions.

Lastly, we note the need to follow this work with research using predictive and econometric methods to build a more solid evidence base for policy making. That said, isolating the impact of a single intervention amidst the ever-changing and crowded landscape of a global market may not be possible and/or may require adaptation or development of new research methods.

Finally, any gains in market efficiency and access to ARVs must ultimately be linked to health outcomes to ensure that the overarching public health goals are achieved. This paper examines relationships between global policies and market dynamics but additional research is needed to better understand relationships between these types of policies and health outcomes (e.g., resistance, treatment failure, progression to 2^nd ^and 3^rd ^line regimens).

## Conclusion

Rapid scale-up in access to ART from 2003-2008 was facilitated by global policies and initiatives that resulted in a fairly efficient global marketplace for older ARVs. However, due to a range of factors, markets for the newly recommended ARVs have been slower to deliver the price reductions and improved formulations seen in the past. WHO Guidelines heavily shape demand, and their relative complexity may help or hinder the achievement of economies of scale in pharmaceutical manufacturing. Certification programs assure ARV quality but can also delay uptake of new formulations. Donor procurement policies, including pooled procurement, may alter ARV market structure by reducing the number of buyers and sellers, rendering the market less competitive in the longer-term and requiring careful monitoring. Improved understanding of ARV markets is required in order to ensure that interventions have their intended impact, i.e. to provide quality-assured ARVs in acceptable formulations at sustainable prices. Global consensus is needed on the ultimate goals of market-based interventions to ensure that short-term gains do not result in detrimental long-term market effects. This will involve clarifying and agreeing on definitions of market efficiency, indicators to monitor market evolution, and methodologies to identify market failures and assess market impacts of policy interventions.

## Competing interests

BW, MK, TB, and SM serve as consultants for UNITAID.

## Authors' contributions

BW designed and coordinated the study, participated in data cleaning and data analysis, and was the lead author on this paper. ED, LS and SM contributed to data analysis and writing of the manuscript. MK and TB contributed to the writing of the manuscript and edited it for important content. JH conducted data analysis and contributed to the writing of the manuscript. All authors read and approved the final manuscript.
